# NeuroTessMesh: A Tool for the Generation and Visualization of Neuron Meshes and Adaptive On-the-Fly Refinement

**DOI:** 10.3389/fninf.2017.00038

**Published:** 2017-06-22

**Authors:** Juan J. Garcia-Cantero, Juan P. Brito, Susana Mata, Sofia Bayona, Luis Pastor

**Affiliations:** ^1^Department of Computer Engineering, Universidad Rey Juan Carlos, Madrid, Spain; ^2^Center for Computational Simulation, Universidad Politécnica de Madrid, Madrid, Spain; ^3^Universidad Politécnica de Madrid, Madrid, Spain

**Keywords:** geometry-based techniques, multiresolution techniques, GPUs and multi-core architectures, compression techniques, bioinformatics visualization

## Abstract

Gaining a better understanding of the human brain continues to be one of the greatest challenges for science, largely because of the overwhelming complexity of the brain and the difficulty of analyzing the features and behavior of dense neural networks. Regarding analysis, 3D visualization has proven to be a useful tool for the evaluation of complex systems. However, the large number of neurons in non-trivial circuits, together with their intricate geometry, makes the visualization of a neuronal scenario an extremely challenging computational problem. Previous work in this area dealt with the generation of 3D polygonal meshes that approximated the cells’ overall anatomy but did not attempt to deal with the extremely high storage and computational cost required to manage a complex scene. This paper presents NeuroTessMesh, a tool specifically designed to cope with many of the problems associated with the visualization of neural circuits that are comprised of large numbers of cells. In addition, this method facilitates the recovery and visualization of the 3D geometry of cells included in databases, such as NeuroMorpho, and provides the tools needed to approximate missing information such as the soma’s morphology. This method takes as its only input the available compact, yet incomplete, morphological tracings of the cells as acquired by neuroscientists. It uses a multiresolution approach that combines an initial, coarse mesh generation with subsequent on-the-fly adaptive mesh refinement stages using tessellation shaders. For the coarse mesh generation, a novel approach, based on the Finite Element Method, allows approximation of the 3D shape of the soma from its incomplete description. Subsequently, the adaptive refinement process performed in the graphic card generates meshes that provide good visual quality geometries at a reasonable computational cost, both in terms of memory and rendering time. All the described techniques have been integrated into NeuroTessMesh, available to the scientific community, to generate, visualize, and save the adaptive resolution meshes.

## 1 Introduction

Understanding the human brain remains one of the greatest research challenges for Science, being one of the most active areas of research. Besides the intrinsic interest in understanding what makes us human, unraveling how the brain works will bring advances in many fields, from revolutionary computing technologies to the development of new treatments for brain disorders. Ambitious initiatives such as the Human Brain Project (EU) (Markram et al., 2011) or the BRAIN Initiative (USA) (Jorgenson et al., 2015) promote the collaborative efforts from multidisciplinary research teams, bringing this goal within reach for the very first time.

Multiple factors are behind the overwhelming complexity of the brain. First, the number of neurons and synapses is huge: it has been estimated that the human brain includes some 10^11^ neurons and 10^15^ synapses (Sporns et al., 2005). Second, the availability of many different techniques for analyzing brain structure and function has resulted in a collection of multilevel descriptions of the brain, coming often from many different perspectives and disciplines. Neuroscience itself can be seen as a set of different subdisciplines that study the brain from complementary points of view (anatomical, physiological, etc.).

Working at microscale, and from a morphological point of view, the acquisition of the anatomy of neurons from stacks of microscopy images can be accelerated using a range of software tools. However, the automation of this process requires the development of automatic segmentation processes, which is a milestone that has not yet been fully achieved. For this reason, acquiring the morphology of neurons usually involves the interactive tracing of neuron elements from microscopy images. This task is carried out by a human operator who typically has to perform operations such as setting parameters, marking sparse control points that describe the neurite trajectories and providing a soma approximation (a 2D contour, or a center and a radius in coarse approximations) before the morphological tracing is finished. This is particularly true with data acquired over recent years and stored in databases such as NeuroMorpho (George Mason University, 2017).

Regarding the visualization of digitized neurons, there are methods that allow the generation of 3D meshes to approximate the neuronal membrane, but the visualization of complex 3D neuronal scenes or large collections of individual neurons poses some challenges to these approximations, requiring special attention. First, the morphological tracings provided by neuroscience laboratories do not include a complete description of all the parts of the neuron. This is especially true in the case of the soma, where a 2D contour is not enough to recover the 3D shape of the cell body. Second, the number of neurons is often quite large, like in modern simulators that use neural models with detailed morphology. In these cases, it should be noted that the geometry of each neuron is unique and far from simple, making the visualization of complex neural scenes a challenging computational problem. Nevertheless, visualizing the scene is mandatory for designing and reviewing simulation scenarios, analyzing results, etc.

This paper presents a multiresolution approach for the 3D visualization of detailed neuron reconstructions, suited for the recovery of data from existing databases and for the visualization of complex neuronal simulation scenarios. The method presented here first generates an initial coarse mesh, from the incomplete descriptions obtained from morphological tracings and then refines it in the graphic card during visualization. In summary, the main contributions of this paper are as follows:

- An improved technique for the 3D reconstruction of the soma, for cases where it had not been previously generated. The method is based on a physical simulation approach that deforms an initial simple shape according to the distribution of the first-order neurites. The deformation is computed using a Finite Element Method (FEM).
- A set of techniques for the generation of low-resolution 3D models of cell membranes that follow the trajectories described in the morphological tracings, incorporating the plausible 3D soma shapes previously generated.
- An on-the-fly adaptive refinement method of the coarse mesh previously generated, making use of the tessellation capabilities of the GPU.
- A first version of the tool that implements the techniques: NeuroTessMesh. This software, publicly available at http://gmrv.es/neurotessmesh, allows meshes approximating neuron morphologies to be generated, visualized, and saved. NeuroTessMesh was developed in C++ and has been released for Linux and Windows operating systems.

The set of techniques presented here constitutes a framework that allows the visualization of neurons recovered from coarse mesh or even incomplete data. Also, it allows the rendering of complex neuronal scenarios, managing the high complexity derived from the intricate geometry and potentially huge number of elements involved. The techniques that have been developed are specifically adapted to the field of neuroscience, taking the compact descriptions of cell anatomies directly provided by neuroscience laboratories as input and incorporating specific techniques for the recovery of 3D shapes that are not completely described. Results show a good trade-off between visual quality and computational cost, both in terms of memory and rendering time.

## 2 Background

The method for mesh generation proposed in this paper has been specifically designed for neuroscience data extracted from biological samples. The procedure for acquiring these samples starts with the staining of individual neurons in thin slices of brain tissue. There are different staining techniques, each of which is specifically suited for particular experiments, and selecting the appropriate method is crucial to ensure a proper acquisition (Parekh and Ascoli, 2013). After any of these chemical staining processes, microscopes are able to capture the neuron morphology, including the somata, dendrites, and axons. Modern techniques such as multiphoton microscopy (Zipfel et al., 2003) automatically generate 3D image stacks of brain tissue, with image planes separated from each other by only a few micrometers.

From these image stacks, it is possible to trace the 3D contour or path of the main components of each neuron to digitally reconstruct the neuron morphology in a manual (Glaser and Glaser, 1990), semiautomatic (Oaks et al., 2016), or totally automatic way (Xie et al., 2010; Peng et al., 2011). The extracted shape and the placement accuracy of the morphological points traced along the neuron contour or path are highly dependent not only on the quality of the obtained image stacks but also on the expertise of the human operator, who is in charge of placing the morphological points within the neurites by manually clicking with a mouse, or by setting the parameters in (semi-)automated algorithms.

The morphological tracing procedures described above define a unique tree structure, with a root node at the cell soma and an ordered sequence of interconnected nodes that defines segments within the original shape of the neurites, also including the neurite thickness at each morphological point. Unlike the neurite tracings, a detailed description of the soma is not commonly stored. Typically, the data stored in these tracings include a unique morphological point placed at the soma center plus the average soma radius, or a set of connected points tracing the 2D projection of the soma contour from a specific point of view (which is clearly not valid for other points of view).

## 3 State of the ART

Although tracings provide essential information, one limitation when visualizing them is that it is not possible to perceive the neurite thickness. Also, visualizing several overlapping segments of the tracing can be ambiguous, since there are no “clues” to indicate which segment is on top of the other. Visualizing their corresponding 3D meshes improves the spatial perception, allowing the user to better perceive relations and how the different neurites relate to one another spatially including their proximity. Also, the neurite thickness and volume are immediately perceived, and a 3D shape of the soma can be viewed. In addition, having a 3D mesh makes it possible to associate values with the neural membrane.

The 3D visualization of digitized neurons presents some problems. If mesh-based methods are to be used for rendering, it is necessary to generate meshes with enough resolution to capture fine detail. However “enough resolution,” is a fuzzy term that might depend on the particular task and user environment in question. This is a common problem in many 3D computer graphics applications, and perhaps the main issue in this case is that the number of neurons to be displayed can increase above any prespecified limit (such as in large-scale simulations using detailed geometric models for neurons). This imposes additional scalability restrictions when attempting to come up with practical solutions. In addition, it is common to find that publicly available collections of 3D neuron reconstructions do not have complete geometrical descriptions, which are necessary to generate the meshes from the available data.

Several software packages, such as Neurolucida (Glaser and Glaser, 1990), Imaris (Bitplane, 2016), NeuroConstruct (Gleeson et al., 2007), NeuGen (Eberhard et al., 2006), and Genesis (Wilson et al., 1988) provide approximations of neuron surfaces but are not focused on the realistic 3D reconstruction of soma shapes, leading to the generation of low quality soma meshes that are not connected with the dendrites. NeuroConstruct, Genesis, and NeuGen approximate the soma with very simple 3D shapes; NeuroConstruct uses a cylinder, and Genesis and NeuGen both use a sphere. In the case of Neurolucida, the soma is approximated with a 2D disk, which is not even saved when exporting the 3D model. More recently approximations such as the toolbox Py3DN (Aguiar et al., 2013) try to achieve more realistic soma reconstructions through geometric approximations. In this case, the tool adapts a set of successive overlapped planes that are generated taking into account the dendritic initial points. However, this toolbox does not connect the generated soma with the dendrites either. Other methods such as Lasserre et al. (2012) are able to obtain a smooth and connected representation of the soma. This method starts from a sphere (made with quads) with a fixed resolution, where the dendrites are generated by quad-extrusion starting from the soma. At the end of the method, a Catmull–Clark subdivision smooths the whole mesh, generating realistic, smooth, and closed meshes. Nonetheless, due to the fixed initial soma geometry, the final shape of the obtained soma continues to be too spherical. Neuronize (Brito et al., 2013) defines a physically based generation method using a mass-spring system. Neuronize generates not only a realistic soma but also a good approximation of important morphological parameters such as the soma volume and area. However, due to the versatility of the mass-spring system, this generation may require complicated fine-tuning of several simulation parameters to achieve an accurate soma reconstruction.

The visualization of complex neuronal scenes requires special techniques for managing the intricate scene geometry. Multiresolution approaches (Clark, 1976; Luebke et al., 2002) have been traditionally used in these kinds of situations, due to their ability to manage different representations of the same objects in a given scene, selecting the most appropriate representation in each case according to different criteria (Luebke et al., 2002). This approach has been followed in methods for neural membrane CPU mesh generation with different levels of detail (Brito et al., 2013), where the mesh resolution is fixed through the specification of the number of sections and cross sections for each segment of the morphological tracing, and (Lasserre et al., 2012), where different levels of detail are obtained through consecutive application of the Catmull–Clark (Catmull and Clark, 1978) subdivision algorithm.

Clark’s classic approximation (Clark, 1976) improves rendering performance, but at the same time, presents some problems, such as huge memory requirements, due to the different representations of each object in the scene. In neuronal scenes, it is not possible to store all the representations in the graphic card memory, due to (i) the vast number of neurons and their complex morphology and (ii) to the constant data transfer from the main memory that are required. Alternative multiresolution techniques (De Floriani et al., 1998) introduce a multitriangulation approach, where a hierarchical model is generated and stored together with the approximation error of each mesh update. This data structure can be queried at runtime to extract a simplified mesh fulfilling some defined restrictions. Another approximation is Progressive Meshes (Hoppe et al., 1993), where a sequence of edge collapse operations is applied over the mesh to simplify the model, recording the sequence applied to allow the original quality to be recovered. These two approximations result in substantial CPU loads to traverse the triangulation and, at the same time, large memory requirements to store the highly detailed meshes. These issues hamper their applicability to complex neuronal scenes.

Classical refinement approximations such as subdivision surfaces (see Shröder et al. (1998) for a survey) reduce memory requirements, since this type of process only requires a coarse mesh to generate high order surfaces over each polygon. The problem with this approximation is that the refinement process generates a huge number of primitives that need to be sent to the graphic card, generating large data transfer and bus bottlenecks. To avoid this, some of these algorithms are being deployed directly in GPUs, such as a hardware evaluation of the Catmull–Clark schemes, proposed in Shiue et al. (2005) or loop subdivision proposed in Kim and Peters (2005). Also a procedural displacement is performed over the new generated vertices, making use of local information in each of the patches being processed, using triangles (Boubekeur and Schlick, 2005) or quadruples (quads) (Guthe et al., 2005). Schwarz and Stamminger (2009) have proposed a unified pipeline through GPGPU techniques. Their proposed framework applies refinement techniques using CUDA and incorporates several tessellation-based techniques from the literature, such as Bicubic rational Bézier patches (Farin, 2002) or Curved Point Normal Triangles (Vlachos et al., 2001), where the refinement is achieved by the construction of Bézier patches over each triangle of a coarse mesh. In this approximation, the construction of each Bézier patch only needs the local values of each triangle (the position and the normal of the vertices) for the refinement. Based on this idea of local refinement, Boubekeur and Alexa (2008) proposed the Phong Tessellation, inspired by Phong (1975), but instead of interpolating the normals, the authors use the plane’s tangent to the mesh vertices to define a curve geometry for each triangle. This last approximation provides a better performance than Curved Point Normal Triangles (Boschiroli et al., 2011); however, the memory access time remains prohibitive, even within the graphic card.

As a consequence of these problems, new stages have been included in the classical GPU pipeline, making the GPU more programmable and avoiding the need for storing each newly generated vertex in the graphic card memory. Geometry shaders can perform some simple refinements through simple tessellation techniques (Lorenz and Döllner, 2008), where the refinement is performed using an incremental multi-pass scheme based on previously refined meshes using precalculated patterns stored in the graphic card memory. However, this stage usually significantly slows down the pipeline, if it needs to manage a large number of geometry primitives (Schnetter et al., 2004). To avoid this problem, new generations of GPUs have added new stages into the classical pipeline to facilitate tessellation tasks. Thus, the pipeline is expanded allowing users to have a precise and easier control of the geometry, making it possible to manage the level of detail of the desired models directly on the GPU. A good overview of these types of techniques can be found in Nießner et al. (2016), where a large number of different examples using hardware tessellation for efficient rendering are analyzed in depth.

The mentioned techniques are oriented toward objects of generic shapes. For neuroscience data, the objects to be modeled have a number of specific features that should be considered early in the technical design process, because they are key for optimizing method performance when dealing with scenes composed of large numbers of neurons. Some examples of how this can be done are presented in the following sections.

## 4 Methods

The main goal of the techniques presented here is the design of efficient representations that:

- Are well adapted to the particularities of the elements that can be found in complex neuronal scenarios (from the point of view of computer graphics).
- Are accurate and faithful with respect to the real baseline data.

**Figure 1:**
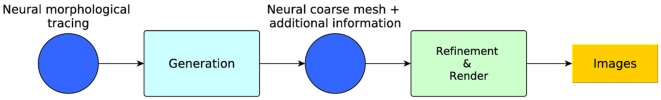
Method overview: generation and refinement modules. From the morphological tracing, a coarse mesh is generated with additional information. A dynamically adaptive LOD refinement is then applied to the coarse mesh for its real-time visualization.

To accomplish this goal, this paper proposes a set of techniques that can be grouped into two modules (Figure 1): the first module takes as input any existing morphological tracings from (possibly real) neurons and generates a coarse, low-poly 3D mesh, together with some additional information which allows the mesh to be used by any application capable of representing 3D meshes. The second module takes the coarse generated mesh with the additional information and performs a view-dependent (or other criterion-dependent) refinement to render it at dynamically adaptive levels of detail (LOD). The following sections describe these modules in detail.

### 4.1 Generation Module

The goal of this module is to generate an initial low-poly mesh that approximates the whole neuron. As previously mentioned, this method is based on existing morphological tracings such as those stored in NeuroMorpho. These tracings usually describe the soma and neurites in different ways: neurites are described by polylines that trace their trajectories, while somata are often barely described (by a 2D contour at most). Therefore, the strategies applied for the mesh generation of these two structures are different, obtaining separated meshes that are merged in a final step.

Regarding somata, the solution presented here for their generation is an improvement on the approach proposed in Neuronize (Brito et al., 2013). The underlying idea is to select an initial simple shape (in our case, a sphere) and simulate the physical deformation this sphere would undergo in the hypothetical case that the neurites attracted the sphere surface toward them, generating an elastic deformation of the sphere. In this paper, the original technique is improved by applying an FEM (Finite Element Method) (Erleben et al., 2005) to simulate the deformation.

**Figure 2:**
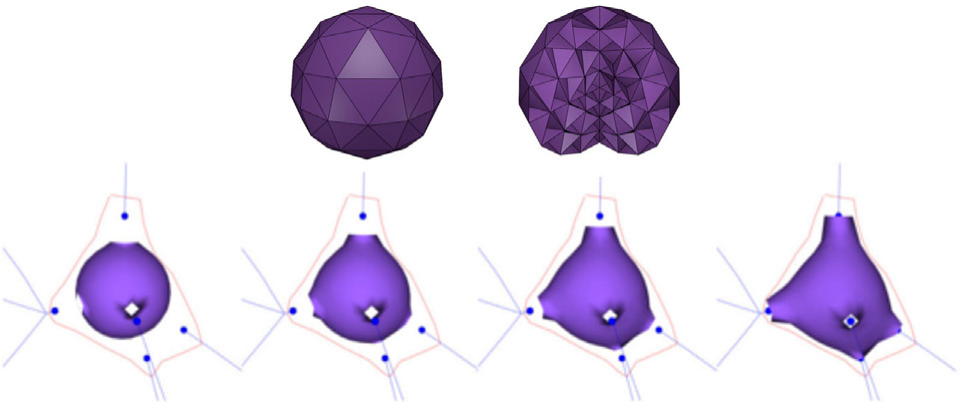
Top images: initial tetrahedral icosphere used in the soma generation process. View of its surface (left image) and of its internal structure (right image). Bottom images: FEM deformation process. During this process, the original icosphere is deformed by forces applied at the dendrites’ insertion points.

Since FEM works on volumetric models, the first step to obtain each soma is to create a volumetric representation of an icosphere. Hence, based on the soma center and radius as provided by the morphological tracings, a tetrahedral mesh is built (Figure 2; top). This volumetric mesh is taken as the initial equilibrium state for an elastic deformation process. The external faces of the tetrahedra form a triangular mesh that represents the surface of the icosphere. However, since quads are more suitable than triangles for subsequent steps of our method, pairs of adjacent triangles are merged into quads. Afterward, the surface quads closest to each neurite are selected, and their vertices are pulled toward the neurite insertion point in the soma. The size of this quad is adapted to match the neurite’s diameter at its starting point. Finally, applying a static linear FEM, the mesh is deformed until the final shape of the neuron soma is generated (Figure 2; bottom).

**Figure 3:**
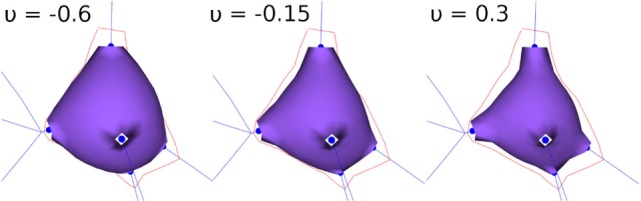
FEM deformation. Variations of Poisson’s coefficient resulting in variations in soma volume.

During the FEM-based deformation stage, variations in the Poisson’s coefficient, *v*, result in different soma deformations and final soma shapes; decreasing the Poisson’s coefficient results in a varying degree of soma swelling, as shown in Figure 3. Note that the Young’s modulus is not modified, since a static linear implementation is applied.

Regarding neurites, the soma quads which are already positioned at the beginning of each neurite define the initial section of their respective neurite. Next, each initial section is extruded between each pair of neurite tracing points, following the neurite trajectory, to approximate the tubular structure of the neurite membrane. In addition, since the changes in neurite directions occur at the traced morphological points, an orientation vector is computed at each one of these points to re-orient the quad section associated with each morphological point.

Three different cases can be distinguished for the computation of the orientation vectors: one associated with standard tracing points (points that have only one child), one with bifurcation or fork joint tracing points (points that have two children), and one with ending tracing points (points that do not have any children).

**Figure 4:**
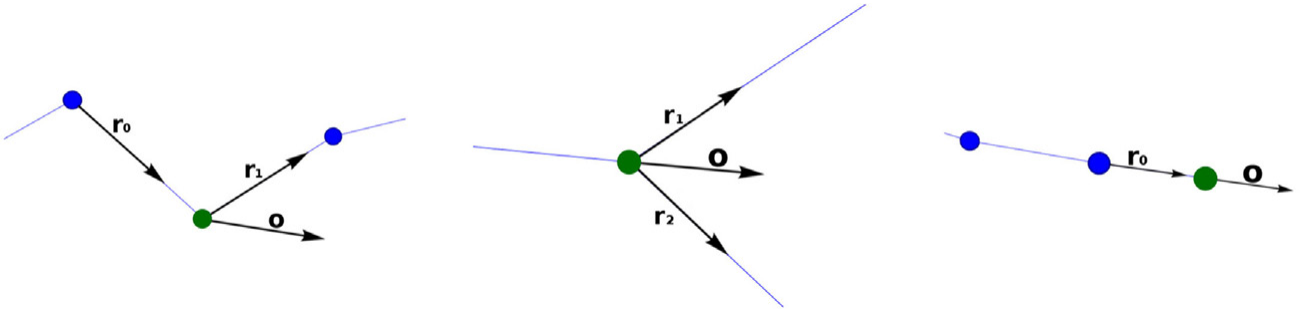
Calculating the orientation vector according to the different types of tracing points. The current point is represented with a green sphere. Left image: standard tracing point. Middle image: bifurcation tracing point. Right image: ending tracing point.

The orientation vector, **o**, of a standard tracing point is the result of adding the vectors **r_**0**_** and **r_**1**_** and normalizing the resulting vector (Figure 4; left), where **r_**0**_** is the unit vector indicating the direction between the parent tracing point and the current tracing point and **r_**1**_** indicates the direction between the current tracing point and the child tracing point.

Computing the orientation vector, **o**, at the bifurcation tracing point is performed in a similar way, but in this case, the unitary vectors **r_**1**_** and **r_**2**_** give the directions defined by the current tracing point and each of its children (consequently, **o**, does not depend on the parent segment orientation vector) (Figure 4; middle). Finally, at ending nodes, the orientation vector, **o**, is equal to the unit vector **r_**0**_** (Figure 4; right).

**Figure 5:**
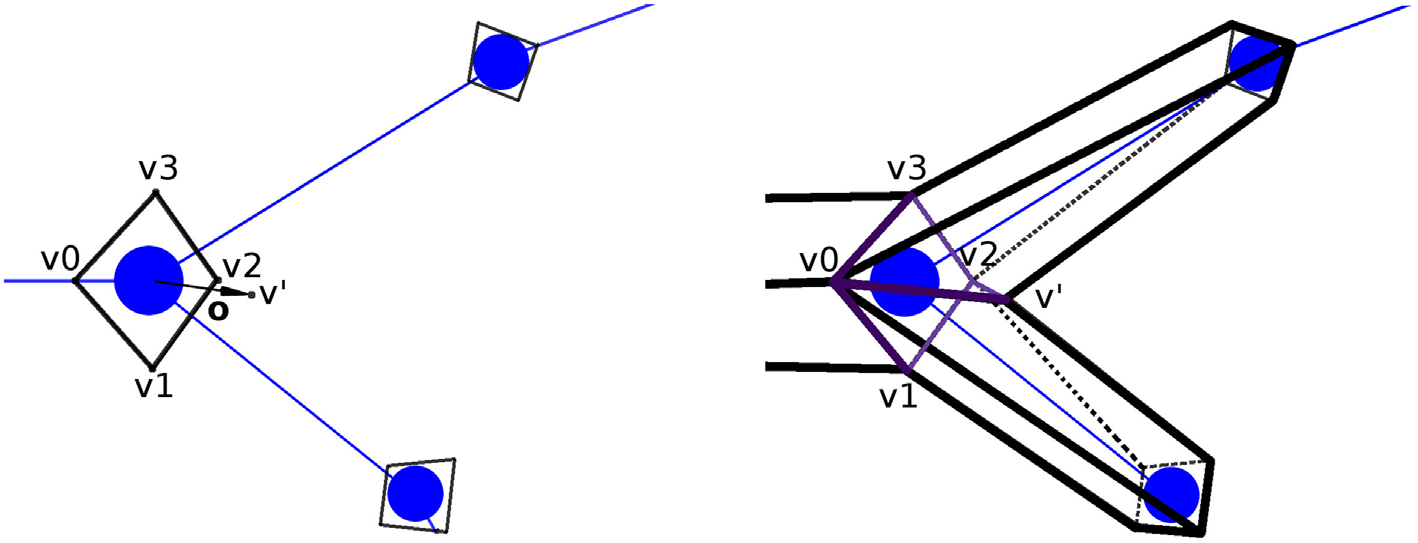
Generation of the extra vertex, v′, at bifurcation tracing points, at a distance equal to the radius of the bifurcation (left image), for its posterior stitching (right image). In this example, the new quads (separated by the bifurcation section-quad diagonal v0–v2) are [v0,v1,v2,v′] and [v0,v′,v2,v3].

Once the orientation vectors have been computed, a section-quad is positioned at each tracing point, oriented according to its orientation vector computed as described above. The section-quad is also scaled according to the radius of the tracing point. In the case of a bifurcation, an extra vertex is introduced to facilitate the stitching of these branches, placing this new vertex at a distance equal to the radius of the bifurcation tracing point in the direction of **o**, its orientation vector (Figure 5; left).

Once the section-quads for the whole neurite have been generated, all the vertices are connected to each other to obtain the neurite quad mesh. The quads connecting these vertices are called lateral quads, to distinguish them from the previously mentioned section-quads. There are two special cases that must be dealt with by this process. First, the union at bifurcations, given v0,v1,v2,v3 as the vertices of the bifurcation quad, and v′ as the extra vertex, two new quads (composed by three of the vertices from the bifurcation quad, and the extra v′ vertex) are created. A plane containing the two children tracing points and the bifurcation point is created. Then, based on this plane, we select the most appropriated of the two diagonals of the section-quad (v0–v2 or v1–v3) separating the bifurcation section-quad vertices into two groups of three vertices, which will be used to create the new quads, together with the extra vertex v′. Hence, the two new quads will be either [v0,v1,v2,v′] and [v0,v′,v2,v3], or [v0,v1,v′,v3] and [v′,v1,v2,v3] (Figure 5; right). Second, the connection at the ending tracing points, where the 4 vertices of its section-quad do not need to be connected to any other vertex, is carried out by connecting these vertices to each other through another lateral quad.

Once the coarse neurites and the soma have been generated, their union is straightforward. This is because the first section-quad of a neurite used for its extrusion was itself a soma-quad indicating the neurite starting point. At this point, the generated polygonal mesh provides a coarse approximation of the cell’s membrane that will be refined in the following stage. In addition to this base mesh, some additional information is kept to guide the subsequent refinement step. Specifically, each vertex of the coarse mesh keeps track of its associated tracing point. As a result, at each vertex, the position, radius, and orientation vector of its associated tracing point can be accessed in the following stage.

### 4.2 Refinement and Render Module

The goal of this module is the generation of higher resolution meshes that yield better approximations of the neuron membrane, by building upon the initial coarse mesh obtained as described above. Regarding somata, their resolution is defined by the resolution of the initial icosphere that is subsequently deformed. With respect to the neurites, they undergo on-the-fly refinement procedures, which take advantage of the hardware tessellation capabilities (tessellation shaders) supported by OpenGL from version 4.0 on; this OpenGL version requires a Radeon HD series 5000, an nVidia GTX series 400 or later series of this graphic card vendors. The tessellation process takes each input patch and subdivides it by computing new vertices together with their associated attributes (Shreiner et al., 2013). This tessellation stage is further decomposed into three substages.

The first substage, the Tessellation Control Shader, determines the number of subdivisions (i.e., subdivision levels) that each geometric patch will go through. The second substage, the Tessellation Primitive Generator, takes as inputs the patch and the subdivision levels defined in the previous substage and subdivides the original patch accordingly. Finally, the third substage, the Tessellation Evaluation Shader, computes the attributes of each new vertex generated by the previous substage, such as vertex positions and so on. It should be noted that since only the first and the third substages are user programmable, the present method only needs to compute the number of subdivision levels and the attributes of the newly generated vertices.

Homogeneous refinements can be reached by setting the same subdivision levels for all the object patches. However, given the overwhelming geometric complexity present in regular neurons, the use of adaptive levels of detail is recommended, allowing the neurites closer to the camera to be refined while the detail for distant areas is kept lower. This distance to the camera can be encoded as a generic importance value associated to each tracing point, and this value could also be used to encode criteria other than distance. Since each vertex keeps track of its associated tracing point, assigning importance values to the tracing points is analogous to labeling each vertex with an importance value.

As mentioned above, the first refinement step involves determining the subdivision levels for each patch (it should be noted that each lateral quad of the coarse mesh will be taken as a single patch). To define the subdivision pattern, two different levels must be taken into consideration: an outer subdivision level and an inner subdivision level. The outer subdivision level determines the number of subdivisions at each edge, requiring therefore four parameters in the case of a quad patch (one for each edge). The inner subdivision level determines the number of subdivisions in each edge direction (longitudinal and traversal), requiring therefore two more parameters (Shreiner et al., 2013).

**Figure 6:**
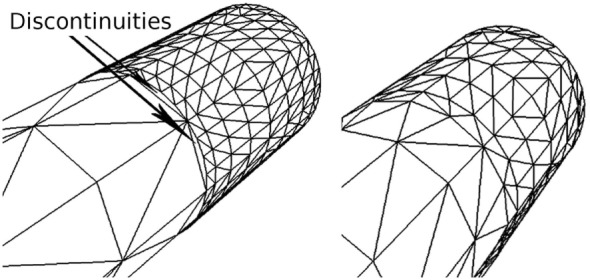
Discontinuities on a refined mesh. The left image shows a refined mesh with discontinuities caused by the difference in contiguous subdivision levels. The right image shows the same mesh refined with our method, in which there are no discontinuities.

Since these levels are set according to the importance of the vertices, discontinuities can occur whenever the importance values of adjacent vertices are very different. For this reason, the outer subdivision level of each edge is computed as a weighted sum of the importance of its two vertices. In addition, both inner subdivision levels have the same value, obtained also as a weighted sum of the importance of the four vertices of the quad. This way of determining the subdivision levels avoids discontinuities on the refined mesh; Figure 6 illustrates a mesh refinement operation that does not prevent discontinuities, which clearly contrasts with the results obtained with the proposed solution, where no discontinuities are created. Note that this method prevents the appearance of discontinuities not only along the neurites but also at the refined neurite-soma connections. Finally, once the subdivisions levels have been defined, the second substage (the Tessellation Primitive Generator) can divide each original patch accordingly.

Finally, the third substage, the Tessellation Evaluation Shader, must compute the position of each new vertex generated by the previous substage. These new vertices are initially positioned on the quad plane to which they belong, so that their final positions are calculated from the homogeneous tessellation coordinates generated in the previous stage: x, the transversal coordinate and y, the longitudinal coordinate.

In our specific case, each new vertex of the patch needs to be displaced to approximate a cylinder, which is the best approximation to the neurite cross section that can be obtained with the available data. In the case of vertices that lie within a section-quad (centered at a tracing point), this operation can be easily performed by displacing each vertex. The displacement magnitude for each vertex should be equal to the radius associated with the tracing point, with the displacement performed in a radial direction from the tracing point. However, the new vertices that do not lie in a section-quad require the computation of a point along the neurite trajectory that behaves as a center point from which the radial directions originate. This process is outlined in the following paragraphs.

**Figure 7:**
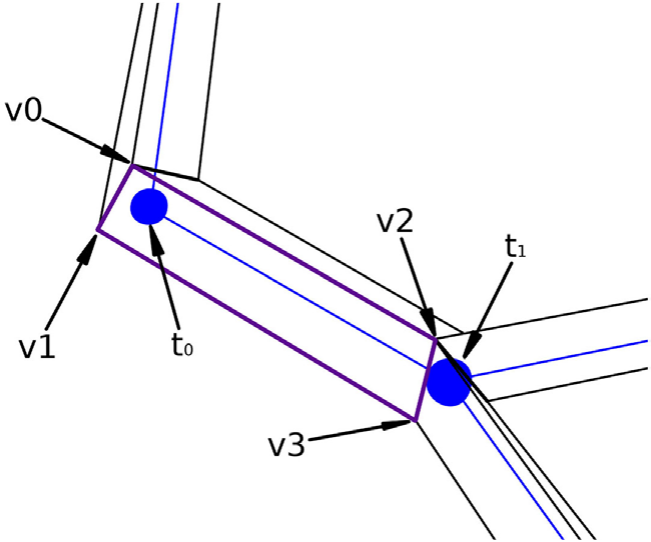
Correspondence between the four lateral quad vertices and their two corresponding morphological tracing points.

Figure 7 presents a portion of a coarse mesh, where a set of four lateral quads represents the union between two morphological tracing points (only one lateral quad, in purple, is depicted). The first two vertices of each lateral quad, v0 and v1, correspond to the first tracing point, *t*_0_, of a tracing segment, and the last two vertices, v2 and v3, correspond to the second tracing point, *t*_1_, of that segment. Because of this, the position of the center, the radius, and the orientation vector associated with the first two vertices of the lateral quad are those of *t*_0_, while the values of *t*_1_ are associated with the last two vertices of the lateral quad.

**Figure 8:**
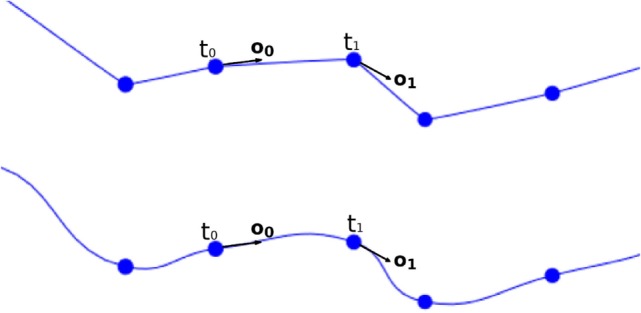
Top image: original neurite path. Bottom image: smoothed path using cubic Hermite spline functions.

For any new vertex, the position of its associated center, as well as the direction and module of the displacement, which will be applied to that vertex, are calculated based on (i) the information of the four vertices of the lateral quad to be tessellated and (ii) the parameters of the two tracing points associated with these four vertices. Therefore, the position of the center associated with any new vertex could be easily computed along the segments that define the neurite trajectory; however, the neuritic paths can be smoothed by interpolating the tracing points with a cubic Hermite spline function. In this case, the position of the center point, *c*, will be computed according to the expression:

(1) c=(2y3−3y2+1)t0+(y3−2y2+y)o0+(y3−y2)o1+(−2y3+3y2)t1,

where *c* is the center to be calculated, *t*_0_ and **o_**0**_** are the position and the orientation vector of the first tracing point, *t*_1_ and **o_**1**_** are the position and the orientation vector of the second tracing point, and *y* is the longitudinal tessellation coordinate. Figure 8 shows the original path of a neurite and the path smoothed using a cubic Hermite spline.

**Figure 9:**
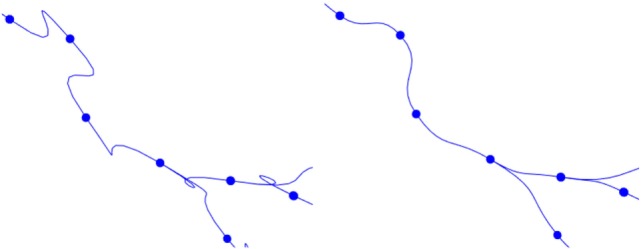
Left image: resulting path when a fixed module for the orientation vectors is maintained. Right image: resulting path when an adaptive module is applied.

This basic formulation of cubic Hermite splines can produce undesired loops when abrupt changes in the orientation vectors of two adjacent tracing points occur. To avoid these artifacts, the module of the orientation vector can be modified, taking into account the distance between the two tracing points of the segment. Figure 9 shows the effects of this improvement.

**Figure 10:**
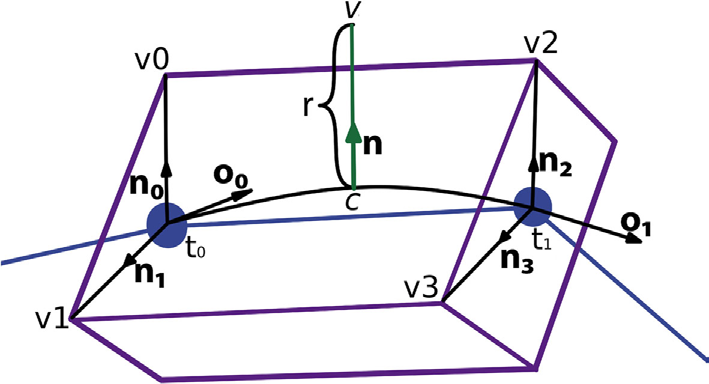
Based on the information associated with the four vertices of the lateral quad and the two corresponding morphological tracing points, the center, *c*, the normal, n, and the displacement, *r*, are calculated to obtain the position of the new vertex, v.

Once the center of the new vertex, *c*, is calculated, the direction of the displacement, **n**, is calculated by performing a bilinear interpolation of the normals of the four vertices of the lateral quad, where these normals represent the radial directions from their associated tracing points. The module of the displacement, *r*, will also be computed by interpolating the radii of the first and second tracing points, *r*_0_ and *r*_1_. Hence, the position of the new vertex, v, is calculated using the following expression, as can be seen in Figure 10:

(2) v=rn+c.

## 5 Results

This paper presents a technique for generating 3D mesh neuron models based on standard, widely used morphological tracings, such as those available in public repositories. The method approximates the cell bodies and the dendritic and axonal arbors in independent procedures that are later merged, resulting in closed surfaces that approximate whole neurons. As described in the previous section, a coarse mesh is the starting point for the method, which dynamically applies subsequent refinement processes to adaptively smooth and improve the quality of the 3D approximation of the cell membrane. This initial coarse mesh presents some desirable properties that make it suitable for visualization and simulation purposes, such as being closed and 2D-manifold. It should be noted that the techniques applied during the mesh generation process guarantee that the traced dendritic and axonal trajectories are preserved, also providing a plausible reconstruction of the soma, specifically built for each cell. This soma reconstruction process is able to recover information that was not recorded when the neuron was traced, which is often the case in existing data repositories.

The following subsections present an evaluation of the quality of the generated meshes and a performance analysis in terms of memory and rendering time.

### 5.1 Soma Reconstruction

In this paper, the original 3D shape of somata is approximated through the deformation of initial spheres, taking into account the anatomy of the dendrites and axon. An initial version of the method was proposed in Neuronize (Brito et al., 2013) using a mass-spring approach. In this new version, the mass-spring method has been replaced by an FEM-based deformation procedure, making control over the deformation results easier, since static FEM implementations only require the configuration of the Poisson’s coefficient, which significantly eases the model generation process with respect to the mass-spring approach used in Neuronize. Figure 3 shows the influence of the Poisson’s coefficient on the volume of the generated soma, obtained after deforming an initial icosphere with 258 vertices and 502 facets.

Concerning the accuracy of the soma reconstructions and their estimated volume (which is of interest for electrophysiological simulations), there were no volume data acquired from digitized neurons, which could serve for quantitative assessment purposes regarding the accuracy of the method. However, to evaluate the method’s accuracy, a visual assessment can give an approximate idea of the reconstruction quality. In addition, measuring the volume of the generated somata can provide some quantitative assessment. For this purpose, soma volumes have been measured and compared with:

- The volume of the real somata, directly estimated from the original data by thresholding, using the Imaris Software (Bitplane, 2016), a very commonly used software program in neuroscience laboratories. These real volumes have been obtained specifically for testing purposes, since they are not usually measured, and have been taken as the ground truth, even though Imaris also introduces volume estimation errors.
- The volume of the reconstructions generated by Neuronize.

**Figure 11:**
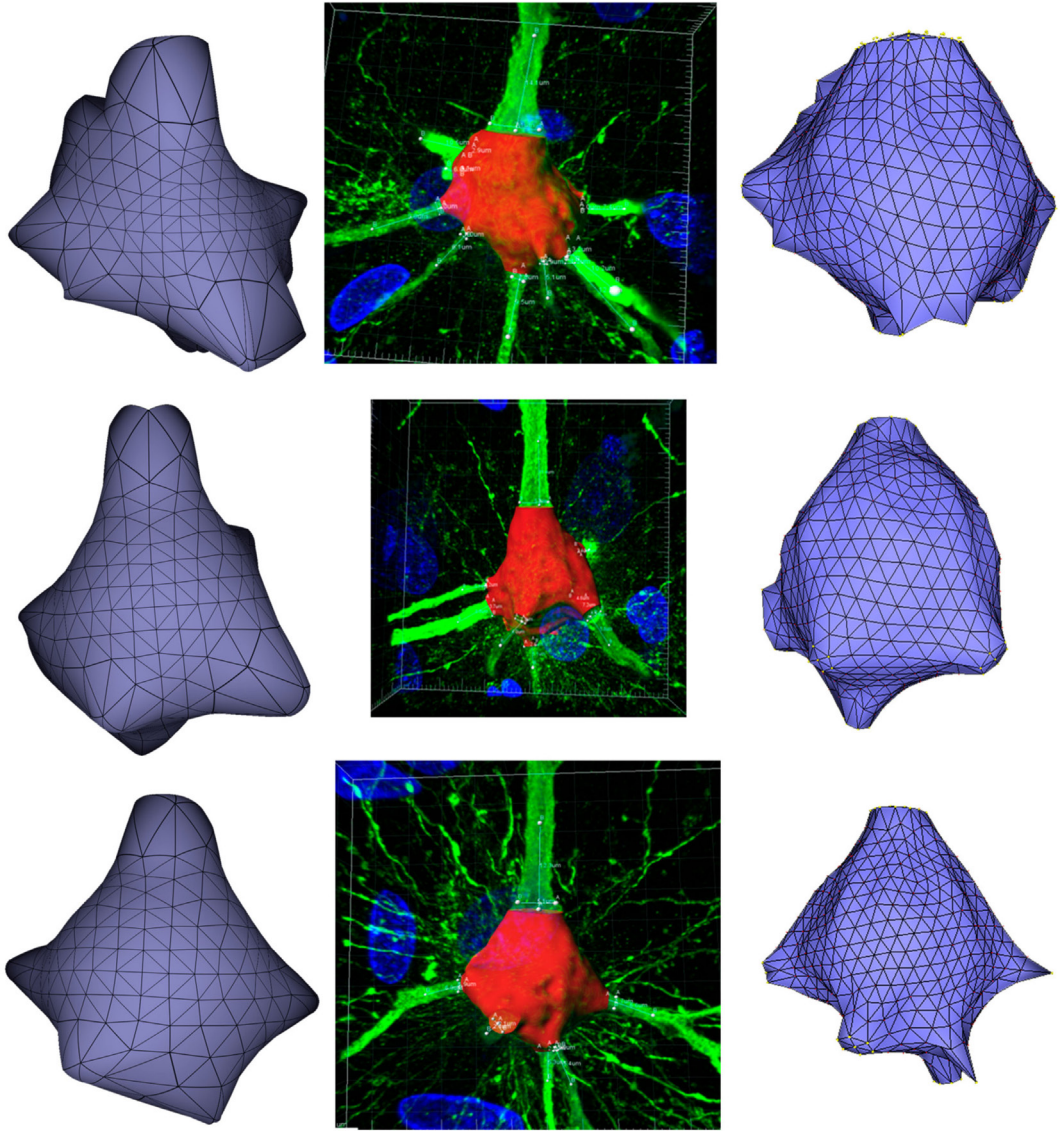
Comparison between the real somata (middle images), the meshes generated with Neuronize (right images), and the meshes generated with our proposal (left images), for three different somata A, B, and C.

Figure 11 presents these results visually, while Tables 1 and 2 present them numerically. Table 1 presents the soma volumes using the different methods, and Table 2 presents the Hausdorff Distance (mean, maximum, and minimum), as a metric to quantify the distance between the real somata and the generated somata (Rockafellar and Wets, 1998). As can be seen, the somata generated with the FEM-based method are closer to the somata obtained with Imaris than the somata generated with Neuronize, according to the Hausdorff Distance metric. In addition, the FEM deformation process returns smoother soma surfaces, avoiding the noisy artifacts that appear on the soma surface when generating it with isosurfaces after thresholding (from Imaris) and with Neuronize. Regarding soma volume, the values obtained with the FEM method correctly approximate the results obtained with Imaris and Neuronize, and the FEM method is much easier to parameterize than Neuronize. However, given the lack of accurate, ground-truth data, it is not possible to state anything specific other than the impression that the results obtained with the proposed method appeared to be largely compatible with those provided by the other methods considered.

**Table 1:** Estimated soma volumes using FEM, Neuronize, and Imaris.

	Volume (μm^3^)		
Soma	FEM	Neuronize	Imaris
A	2,513.8	2,511.85	2,734.8
B	3,473.6	3,516.9	3,618.9
C	1,869.9	1,958.4	1,862.7

**Table 2:** Hausdorff distance (mean, maximum, and minimum) between the real somata, obtained with Imaris, and the generated somata, using Neuronize and the FEM-based method.

	Hausdorff distance (μm)					
Soma	Imaris–FEM			Imaris–Neuronize		
	Mean	Maximum	Minimum	Mean	Maximum	Minimum
A	0.7721	1.6521	0.0003	0.8735	1.6512	0.0011
B	0.8285	1.8180	0.0032	0.9171	1.8196	0.0059
C	0.6482	1.5417	0.0006	0.7819	1.5415	0.0015

### 5.2 Neurite Reconstruction

The neurite reconstruction process presented in this paper guarantees that the reconstructed neurites preserve the original morphological point positions and diameters, as extracted from the original tracings. This is not only the case for the coarse mesh reconstruction but also for the refined meshes generated on the fly, using a procedure that creates very high resolution meshes with low memory penalties.

**Figure 12:**
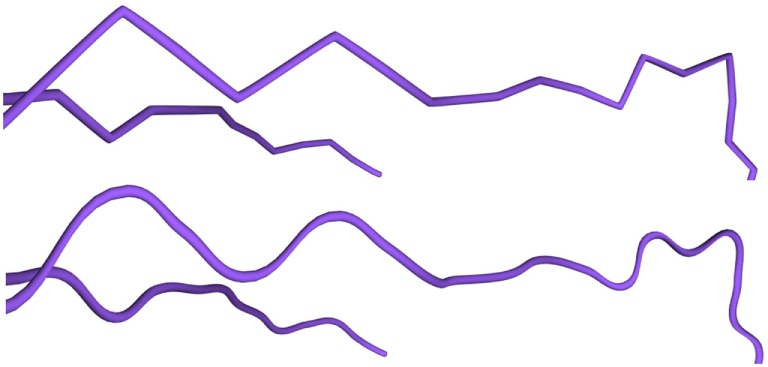
Trajectory smoothing. Top image: refinement method applied to dendrites without any trajectory smoothing. Bottom image: refinement applied to the same dendrites using the Hermite spline-based method proposed in this paper.

The neurite refinement process has been specifically designed for constructing cylindrical shapes from the initial low resolution mesh, since the data available in morphological tracings do not facilitate other approximations for neuron processes beyond those based on generalized cylinders. The reconstructed cylindrical shapes are always crack-free, due to the intrinsic characteristics of the proposed hardware tessellation process, even when the mesh includes sections with different degrees of resolution. In addition, to increase the visual quality of the generated meshes, the trajectories of the morphological tracings can be smoothed using a spline based technique. In this way, the neurite paths become more even, avoiding abrupt trajectory changes that are not found in biological samples but that are created during the morphology acquisition process, as can be seen it Figure 12. It should be noted that, even after smoothing, the original morphological points of the neuronal tracings are always maintained.

**Figure 13:**
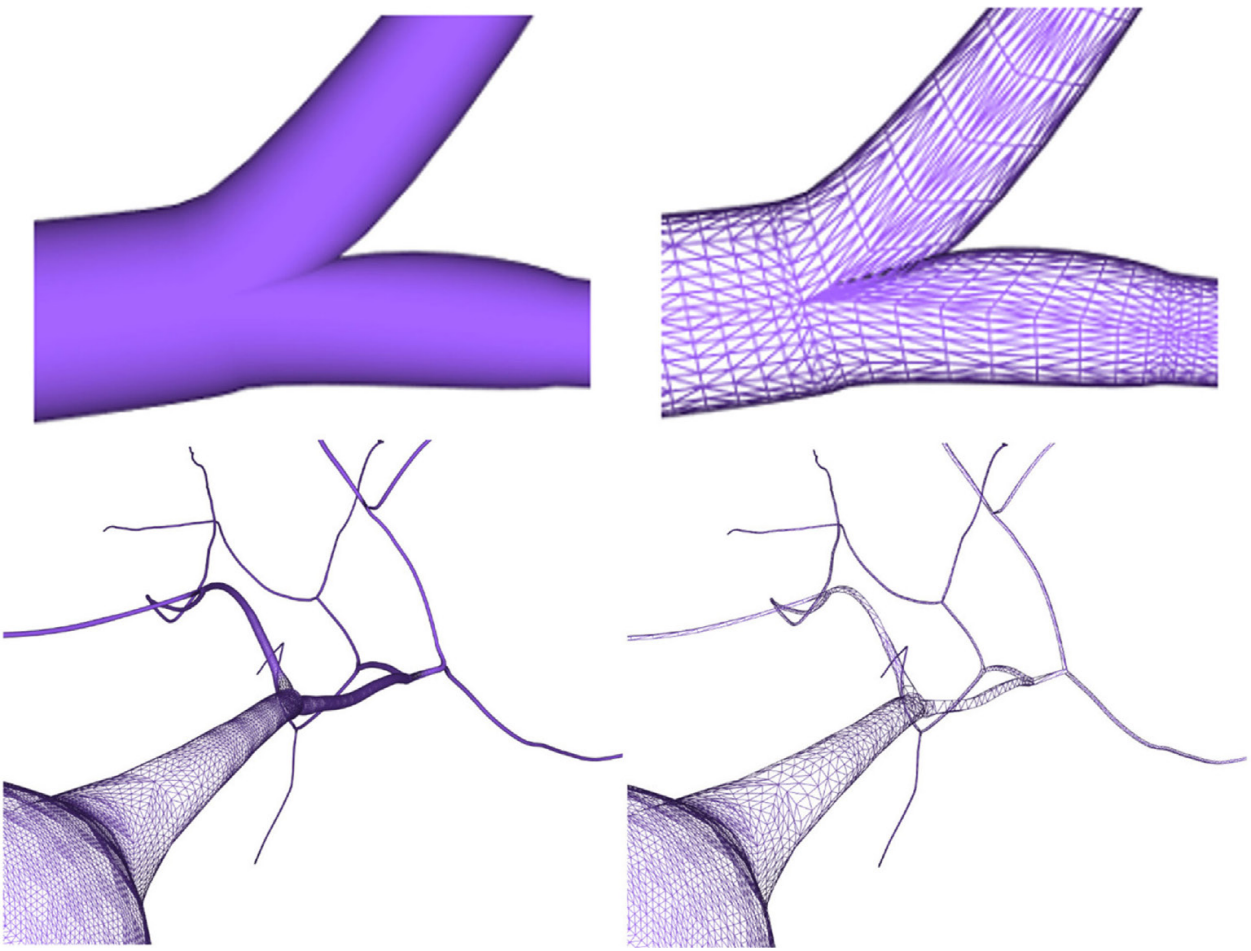
Top images: close view of a generated neurite fork junction rendered in shading mode (left image) and wireframe mode (right image). Bottom images: close view of soma–neurite junction for neuron meshes with homogeneous level of detail (left image) and adaptive level of detail based on the camera distance (right image).

After generating the different neuron component meshes, they need to be connected to assemble the whole modeled neuron. The connection strategies used here were designed for providing neurites with smooth and continuous meshes, taking special care with the connections at neurite bifurcations and at the soma. The method presented in this paper generates smooth unions of mesh components regardless of the resolution of the final mesh, increasing the overall quality of the resulting mesh. Figure 13 (top) shows a junction in a neurite bifurcation in detail, while Figure 13 (bottom) shows a soma-neurite junction.

### 5.3 Performance Analysis

**Figure 14:**
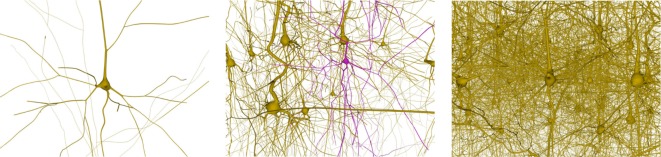
Neuronal scenes used to evaluate the proposed method. Left image: a simple scene with just one neuron. Middle image: a scene with 10 different neurons showing one that has been selected. Right image: a more complex scene with one hundred neurons.

This section analyzes the graphic card memory consumption and the rendering time required for visualizing neuronal scenes measured in frames per second (FPS) using the proposed techniques. Four different scenarios were generated: one, ten, thirty, and one hundred neurons (see Figure 14). Note that in the scene with one hundred neurons, only thirty different morphologies, which are replicated more than once, are stored. The reason for this replication is that, otherwise, the graphic card memory consumption in the pre-refined case would be prohibitively high, and using this approach makes it feasible to compare techniques. These scenarios were rendered using following three different methods:

1. Meshes pre-refined at a fixed resolution and rendered following the standard pipeline. These pre-generated meshes were stored in the graphic card memory, and the time to transfer them from the CPU to the GPU was not computed.
2. Coarse mesh generation and homogeneous refinement following the proposed approach. The coarse mesh was stored in the graphic card memory, and the time needed to transfer it from the CPU to the GPU was not computed.
3. Coarse mesh generation and adaptive refinement according to the distance to the camera, following the proposed approach. The coarse mesh was stored in the graphic card memory, and the time needed to transfer it from the CPU to the GPU was not computed.

**Figure 15:**
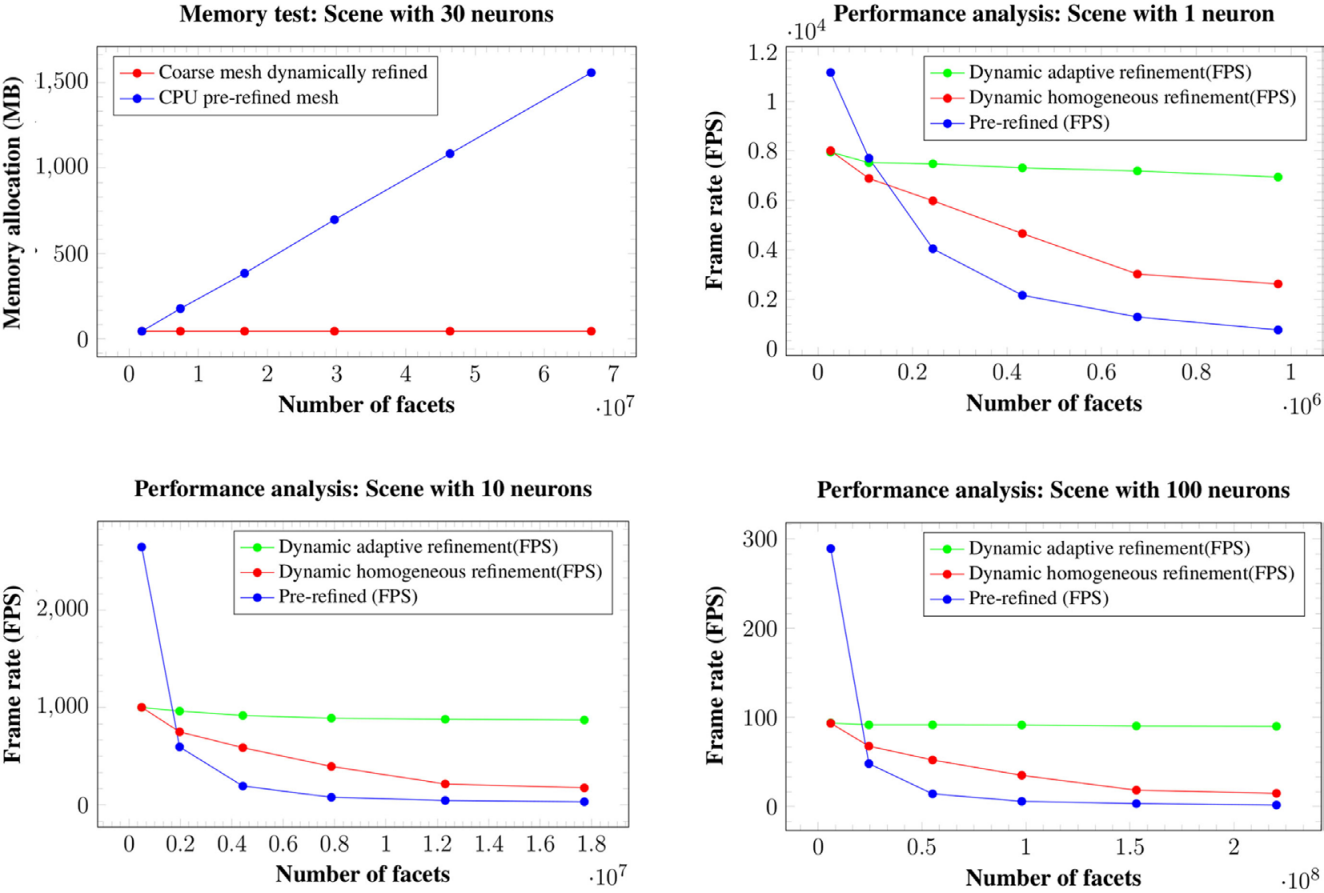
Memory consumption for the 30 neurons scene at 6 different levels of refinement (top left image). Changes in the level of refinement are represented by the dots in the graphics. Both our homogeneous and our adaptive refinement approaches (in red), even including the extra data necessary to apply the refinement, require less memory than storing the pre-refined mesh in the graphic card (in blue). Performance in FPS for the scenes with a single neuron (top right image), ten neurons (bottom left image), and one hundred neurons (bottom right image). Changes in the level of refinement are represented by dots.

Methods 1 and 2 visualize scenes with the same number of polygons on the screen, but method 2 requires less graphic card memory, since it only requires the coarse neurons meshes, that are refined on-the-fly until they achieve the same quality as the pre-generated meshes. Method 3 allows evaluation of the benefits of adapting the resolution according to the distance to the point of view, and therefore lowering the polygon count while maintaining a good visual quality. For all the methods, the frame rate in FPS and the total memory consumption in the graphic card, including the storage of the extra data used for the adaptive refinement, were measured. All results were obtained in a Pentium i7 3.30 GHz with 8 GB of RAM and a Geforce 960 GPU with 4 GB of video memory and a viewport of 600 × 600. All tests were performed using the OpenGL Shading Language. Figure 15 shows the results for the four different scenes analyzed.

Analyzing the memory consumption, it can be seen that in all cases, both the proposed homogeneous and adaptive refinement approaches require less memory than storing the fully refined meshes, despite the fact that there are extra data included for the purposes of applying the refinement. The differences are such that the proposed approaches allow high quality visualization of dense scenes with one hundred neurons, which are impossible to render using pre-refined neurons stored in the graphic card. Moreover, refining the mesh using the tessellation hardware of the GPUs means that the amount of memory required to store a particular scene is constant and independent of the final refinement level, making the method more scalable for high neuron counts and highly refined meshes.

In terms of frame rates, the direct rendering of pre-refined meshes is only faster than the methods proposed here when no refinement is applied, that is, when working at the first level of detail. For the rest of the cases, whenever a refinement pattern is applied (either homogeneously or adaptively), the frame rate is always higher using hardware tessellation than reading the same geometry directly from the graphic card memory. This is because of the high latency of the graphic card memory compared with the intense arithmetic power derived from its massive multi-core architecture.

Moreover, reading the geometry directly from the graphic card memory has a higher penalization in consecutive subdivision levels when compared with the hardware tessellation approach, where the adaptive refinement scales much better in successive refinement levels than the other two methods. For example, in the scene with 100 neurons, from levels of detail of 1 to 6, where the complexity of the geometry becomes 36 times higher, the adaptive refinement has an FPS penalization of only 1.9 times, whereas the homogeneous refinement achieves an FPS penalization of 4.7 times, and the pre-refined meshes obtain a penalization 90 times higher.

Finally, the adaptive refinement approach is not only 2.4 times faster than the homogeneous refinement but also 12.5 times faster than using pre-refined meshes stored in the graphic card memory for the four proposed scenes. From these figures, it is possible to state that the proposed method, using an adaptive refinement approach, scales much better than the other methods assessed, and our method therefore facilitates the interactive exploration of dense, complex neuronal scenes.

## 6 Conclusion and Future Work

The analysis of neuronal systems will benefit from the development of new computational tools that facilitates the exploration of the data gathered by neuroscience laboratories. Visualizing the anatomy of complex sets of neurons can be of great interest, not only for their analysis from a morphological point of view but also as an underlying process for the exploration of electrophysiological simulations or connectivity in neuron networks. Continuous improvements in computing power are leading to changes in the way simulations are carried out in computational neuroscience. In recent years, this has resulted in ever increasingly complex simulations using full neuron anatomy models, instead of the point-neuron models that have traditionally been used for large-scale simulations. In a parallel trend, the improvements in microscopes and laboratory techniques are allowing neuroscience laboratories to gather larger and larger sets of neurons, at increasing levels of resolution.

This paper presents a domain-specific set of techniques for the generation and visualization of neuronal scenes, lowering the computational costs derived from the high complexity of neuronal data, while still providing a good approximation of the real anatomy of the cells. In addition, the techniques presented here allow for the reconstruction of models from previously acquired neurons stored in repositories such as NeuroMorpho.

The direct use of morphological tracings as the input description for the developed techniques bridges a gap between neuroscience and computer graphics. In addition, morphological tracings give a compact description of neuron geometry that can be further deployed in standard polygonal meshes suitable for use in the field of computer graphics or in detailed-geometry simulations. In this regard, the generated meshes have some desirable properties such as being closed and 2D-manifold.

The proposed multiresolution visualization relies on the initial generation of a coarse mesh that approximates the neural membrane. This coarse mesh can be easily refined later on, either homogeneously or according to any criteria such as distance to camera.

Regarding the first stage (coarse mesh generation), the novel method applied for the reconstruction of the 3D geometry of the soma from the incomplete descriptions provided by the morphological tracings achieves promising results. Most of the existing tools do not deal with the generation of the 3D shape of the soma; the FEM deformation model improves the results obtained in Neuronize (Brito et al., 2013), by making it easier to parameterize and by generating a smoother membrane surface. The final neuronal model presents seamless connections between the soma and the neurites, and smooth trajectories even in fork joints.

The second stage (refinement and render) takes advantage of the coarse mesh properties that allow an easy correspondence between the mesh vertices and the tracing points of the morphological description. Using this approach, some additional and easy-to-compute information can guide the positioning of the vertices in the Tessellation Evaluation Shader.

The performance of our approach, compared with the rendering of pre-generated meshes, is clearly better both in terms of rendering times and memory requirements. It should be pointed out that in our current implementation the generation of the coarse mesh is performed in the CPU, while refinement is achieved in the graphic card. The designed techniques are suitable to be fully implemented in the GPU (both the generation of the coarse mesh and its refinement), thereby avoiding the need to transfer meshes from CPU to GPU and reducing the computational time of this initial coarse mesh.

The work presented here may be extended in different ways. The implementation of the whole process in the GPU is the most straightforward. The generation of the soma shape can also be improved by using the 2D contour (when available) either to guide the deformation process or as a final step that makes it possible to fit the generated 3D shape into this extracted 2D contour.

Dendritic membrane could also be improved by adding spines, which could be refined during visualization, following a similar approach to the one presented in this work. Regarding the refinement process, it is not difficult to incorporate criteria other than the distance to the camera, to achieve adaptively refined meshes.

The generated meshes are appropriate for visualization purposes; however, they also have some desirable properties (like being closed and manifold) that could make them suitable for simulation, for example, by mapping the electrophysiological properties to the cells membrane.

Finally, the presented techniques have been designed to work with neuronal data, but they could also be useful in other fields where there are filiform structures or biological elements with incomplete descriptions of some of their parts.

## Author Contributions

JG-C, JB, SM, SB, and LP designed the techniques. JG-C and JB implemented the demos. All the authors participated in writing the paper.

## Conflict of Interest Statement

The authors declare that the research was conducted in the absence of any commercial or financial relationships that could be construed as a potential conflict of interest.

## Supplementary Material

video S1 NeuroTessMesh: an overview.
